# Coronary plaque characteristics and epicardial fat tissue in long term survivors of type 1 diabetes identified by coronary computed tomography angiography

**DOI:** 10.1186/s12933-019-0861-x

**Published:** 2019-05-04

**Authors:** Mona Svanteson, Kristine Bech Holte, Ylva Haig, Nils Einar Kløw, Tore Julsrud Berg

**Affiliations:** 1 Department of Radiology and Nuclear Medicine, Oslo University Hospital, Oslo, Norway; 2 Institute of Clinical Medicine, Faculty of Medicine, University of Oslo, Oslo, Norway; 3 Department of Endocrinology, Morbid Obesity and Preventive Medicine, Oslo University Hospital, Oslo, Norway; 4 The Norwegian Diabetics’ Center, Oslo, Norway

**Keywords:** Diabetes type 1, Atherosclerosis, Epicardial adipose tissue, Computed tomography

## Abstract

**Objectives:**

The aim was to assess coronary atherosclerosis, plaque morphology and associations to cardiovascular risk factors and epicardial adipose tissue (EAT) in patients with long duration of type 1 diabetes mellitus (T1DM).

**Materials and methods:**

Eighty-eight patients with ≥ 45 year T1DM duration and 60 controls underwent coronary CT angiography (CCTA) for evaluation of coronary artery plaque volume (total, calcified or mixed/soft), coronary artery calcification score (CAC) and EAT.

**Results:**

Plaques were detected in 75 (85%) T1DM patients and 28 (47%) controls, p < 0.01. Median (interquartile range) plaque volume (mm^3^) in T1DM vs. controls was: 21.0 (1.0–66.0) vs. 0.2 (0.0–7.1), p < 0.01 for calcified, 0.0 (0.0–8.7) vs. 0.0 (0.0–0.0), p < 0.01 for soft/mixed and 29.5 (3.9–95.8) vs. 0.4 (0.0–7.4), p < 0.01 for total plaque volume. Median CAC was 128 (13–671) vs. 1 (0.0–39.0), p < 0.01 in T1DM vs. controls. Median EAT volume did not differ between the groups; 52.3 (36.1–65.5) cm^3^ vs. 55 (38.3–79.6), p = 0.20. No association between CAC or plaque volumes and EAT were observed. Low time-weighted LDL-cholesterol and HbA1c for 30 years were associated with having plaque volume < 25th percentile, OR (95% CI) 0.18 (0.05–0.70), p = 0.01 and 0.45 (0.20–1.00), p < 0.05, respectively. Time-weighted LDL-c was linearly associated with CAC (beta 0.82 (95% CI 0.03–1.62), p = 0.04) and total plaque volume (beta 0.77 (95% CI 0.19–1.36), p = 0.01).

**Conclusion:**

Long-term survivors of T1DM have a higher prevalence of coronary atherosclerosis compared to controls. Low LDL-cholesterol and HbA1c over time have a protective effect on coronary atherosclerosis. EAT volume was not associated with coronary atherosclerosis in T1DM patients.

## Introduction

Patients with type 1 diabetes mellitus (T1DM) have an increased risk of cardiac events, and coronary atherosclerosis increases the risk substantially [1]. Assessment of plaque morphology is important since non-calcified plaques are more likely to result in acute coronary syndrome than the more stable calcified plaques [2]. Plaque morphology has been shown to predict coronary events in type 2 diabetes mellitus (T2DM) [3]. Due to different pathogenesis between T1DM and T2DM, similar studies should also be conducted in patients with T1DM. Furthermore, epicardial adipose tissue (EAT) has gained increased interest due to reported associations with coronary atherosclerosis [4], and suggested linked to inflammation and early development of atherosclerosis [5].

Coronary computed tomography angiography (CCTA) has evolved as a non-invasive imaging technique for evaluation of stenoses in the coronary arteries, but it is also widely used in quantitative plaque assessments [6]. Evaluation of EAT volumes can also be performed on the same images [7]. Unenhanced coronary artery calcification (CAC) score has a prognostic value for cardiac events in asymptomatic individuals [8], but additional contrast-enhanced CCTA has shown to improve the risk-stratification in asymptomatic patients with both T1DM and T2DM [9].

We have previously reported on a higher prevalence of undiagnosed coronary heart disease among patients with a very long duration of T1DM compared to sex- and age-matched controls [10]. However, there is limited evidence on the morphology, extent and severity of the coronary plaques in T1DM patients versus persons without diabetes [11]. In the present study we have included a population of patients with a long duration of T1DM (> 45 years) in order to identify factors associated with coronary atherosclerosis in a group of long-term survivors. This information may widen the understanding of the possible impact of long-term glycemic control on the morphology of coronary atherosclerosis, and the improved understanding of survival may be important in the management of these patients. Furthermore, a lack of association between CAC and EAT has earlier been reported in T1DM patients [12]. Due to diverse evidence regarding EAT [13], there is a need for complementary evaluation of possible associations with the atherosclerotic characteristics.

The aims of the present study were therefore to (i) assess the morphological characteristics of coronary atherosclerosis by CCTA, (ii) to evaluate the associations between CCTA variables with risk factors for coronary atherosclerosis and (iii) to evaluate differences in epicardial adipose tissue (EAT) volumes and associations with coronary atherosclerosis in patients with long-term T1DM compared to controls.

## Materials and methods

### Patients and study design

The cross-sectional Dialong study of long-term survivors of T1DM was conducted in 2015/2016. As previously described, a chart review of the diabetes participants from the previous 2–4 decades was performed, resulting in long-term longitudinal weighted variables of glycated hemoglobin (wHbA1c), low density lipoprotein cholesterol (wLDL-c) and systolic blood pressure (wSBP) [10]. These measurements were available from 1980, and were calculated as previously described [10, 14]. All the patients with T1DM diagnosed ≤ 1970 attending a state-funded specialised T1DM clinic; the Norwegian Diabetics’ Centre (NDC) in Oslo, Norway were invited. Hundred-and-three patients joined the coronary artery disease substudy. Participants without earlier diagnosed coronary heart disease and eGFR > 45 were referred to CCTA, resulting in 88 participants with T1DM for ≥ 45 years completing the CCTA. The sex and age matched control group undergoing CCTA (n = 60) consisted of healthy, invited spouses/friends of the participants with T1DM. The regional ethics committee approved the study (project no. 2014/851) and all participants signed an informed consent.

### Image acquisition

All examinations were performed on a 128-slice Dual Source Somatom Definition FLASH CT-scanner (Siemens Healthcare, Erlangen, Germany). An unenhanced scan was conducted for the evaluation of coronary artery calcification (CAC). If tolerated, beta blockage (5–20 mg metoprolol, Seloken®, Astra Zeneca) was used to reduce the heart rhythm and Nitroglycerin 0.4 mg (Nitrolingual®, Pohl-Boskamp, Hohenlockstedt, Germany) was administered sublingually. The scan protocol for the CCTA was chosen in concordance with the achieved heart rate as previously described [10]. The contrast media Omnipaque™ 350 mg/mL (GE Healthcare, Princeton, New Jersey) was used for all examinations.

### Image analyses

Image analyses were performed on a Philips Workstation (Intellispace v5, Philips Healthcare, Cleveland, Ohio, USA) with dedicated software (Comprehensive Cardiac, Plaque Analysis, Philips Healthcare, Cleveland, Ohio, USA). Images were assessed using a modified 17-segment American Heart Association model [15]. All segments with a diameter > 1.5 mm and subjectively sufficient image quality were included in the analyses. CAD was defined as presence of any plaque. CAC was calculated with the Agatston method [16]. The plaque volume (mm^3^) was calculated for each plaque differentiated on plaque morphology. Plaques were categorized as calcified when ≥ 90% and soft when ≤ 10% of the volume had a density of > 130 Hounsfield units (HU). All other plaques were defined as mixed plaques [17]. The total plaque volume, total calcified volume and total mixed/soft plaque volume were calculated for each patient. The soft and mixed plaque volume was calculated together for statistical purposes due to small amounts of soft plaques.

The extent and severity of CAD was assessed by the segment involvement score (SIS) and the segment stenosis score (SSS). SIS was calculated for assessment of extent as the number of segments with plaque involvement (range 1–17). SSS was calculated for assessment of the severity of the stenosis. Each segment was scored (grading 1–4) according to the Society of Cardiovascular Computed Tomography’s recommended stenosis grading, based on luminal narrowing; Grade 1: 1–29% stenosis; Grade 2: 30–49% stenosis; Grade 3: 50–69% stenosis and Grade 4: 70–100% stenosis, with a total possible SSS of 0–68 [18].

EAT was evaluated from the unenhanced CT images using SliceOmatic5.0 (TomoVision, Magog, Canada). All tissue with a density between − 190 and − 30 Hounsfield units’ values within the pericardial sac was defined as EAT. All 2.5 mm axial slices were assessed, with the upper limit starting at the right coronary artery and bottom limit at the apex of the heart. Two independent readers analyzed a 30% random selection of the T1DM examinations, and similar for evaluation of intrarater variability.

### Statistical analyses

Descriptive data are presented with numbers (%) for dichotomized variables and mean ± SD for normally distributed characteristics or median, interquartile range (IQR) if not normally distributed. Independent samples t-test or *X*^2^ was used to compare variables among groups. Non-normally distributed variables were log-transformed before conducting the analyses.

Correlation between CCTA measurements and clinical variables was assessed by Spearman’s rho. Linear regression was used to adjust for confounders. Variables with not normally distributed residuals were natural log (ln)-transformed. To solve problem of zero values we added one to each measure before transformation (log (X + 1)). Variables with a correlation of ≥ 0.2 or of special clinical relevance were included in the model, and a backwards approach was chosen. Tested variables included: age, sex, family history of coronary heart disease, smoking, hyperlipidemia, use of statins, retinopathy, persistent albuminuria, angina, waist circumference, systolic BP, diastolic BP, pulse pressure, wHba1c, wLDL-c, HDL-c, triglycerides, SR, CRP, troponins and proBNP. Models were checked by plots of residuals vs. predicted values. The 25th percentile of the total plaque volume was evaluated in a logistic regression analysis for the assessment of associations to a low plaque burden. All regression analyses were performed separately of the T1DM group and the controls due to lack of longitudinal variables in the control group. Inter-and intrarater variability were determined by the intraclass correlation coefficient (ICC). All statistical analyses were performed using IBM SPSS Statistics for Windows, Version 25.0. Armonk, NY: IBM Corp.

## Results

Table 1 shows the clinical characteristics which partly have been published [10]. Briefly, age and sex were comparable between the groups. The T1DM-patients had higher heart rates, systolic blood pressure, pro-BNP, HDL-c, lower LDL-c and a higher use of statins compared with controls. Other traditional risk factors such as hyperlipidemia and smoking were equally distributed between the groups.

**Table 1:** Patient characteristics

	T1DM-patients (n = 88)	Controls (n = 60)	p-value*
Age (years)	61.3 ± 7.1	62.3 ± 6.8	0.38
Female, n%	47 (53.4)	34 (56.7)	0.70
Body mass index (kg/m^3^)	25.8 ± 3.9	25.5 ± 4.2	0.69
Waist circumference (cm)	90.3 ± 13.2	89.1 ± 12.2	0.55
Previous CVD, n%	6 (6.8)	2 (3.3)	0.36
Angina, typical	2 (2.3)	0 (0.0)	0.49
Angina, atypical	21 (24.1)	14 (23.3)	
No angina	64 (73.6)	46 (76.7)	
Systolic blood pressure (mmHg)	146 ± 19.8	137 ± 19.3	< 0.01
wSystolic blood pressure (mmHg)	130 ± 10.6		
Diastolic blood pressure (mmHg)	75.3 ± 8.4	81.7 ± 9.7	< 0.01
Pulse pressure	71.6 ± 16.1	55.0 ± 14.1	< 0.01
Heart rate (bpm)	68 ± 10.3	62 ± 9.3	< 0.01
Hypertension^a^, n%	23 (26.4)	11 (18.3)	0.25
Hyperlipidemia^b^, n%	27 (31.0)	12 (20.0)	0.17
Family history of CVD, n%	10 (11.5)	13 (21.7)	0.05
Daily smokers, n%	5 (5.7)	6 (10)	0.62
Ex-smokers, n%	34 (38.6)	22 (36.7)	
pro-BNP (ng/L)	104.9 ± 110.1	67.4 ± 51.3	< 0.01
eGFR	85 ± 19.2	82 ± 12.8	0.18
Statin use, n%	40 (45.5)	6 (10.0)	< 0.01
HDL-c (mmol/L)	2.1 ± 0.5	1.8 ± 0.5	< 0.01
Statin years	2.8 ± 4.3		
LDL-c (mmol/L)	2.8 ± 0.8	3.9 ± 1.0	< 0.01
wLDL-c (mmol/L)	2.9 ± 0.6		
Triglycerides (mmol/L), median (IQR)	0.77 (0.39–2.85)	0.93 (0.52–2.96)	< 0.01
HbA1c (mmol/mol)	7.4 ± 0.81	5.4 ± 0.28	< 0.01
wHbA1c (mmol/mol)	7.9 ± 0.83		

Data are presented as mean ± SD unless otherwise stated

*T1DM* type 1 diabetes mellitus, *CVD* cardiovascular disease, *NT-proBNP* N terminal-pro B-type natriuretic peptide, *eGFR* estimated glomerular filtration rate, *HDL-c* high density lipoprotein-cholesterol, *LDL-c* low density lipoprotein cholesterol, *wLDL-c* weighted low density lipoprotein cholesterol, *HbA1c* glycated hemoglobin, *wHbA1c* weighted glycated hemoglobin

* Independent samples t-test

^a^Hypertension: previous documented hypertension in the chart or from relevant discharge letters, based on readings with sBP > 140 and/or dBP > 90

^b^Hyperlipidemia: documented hyperlipidemia or a previous total cholesterol reading of > 6.2 or LDL > 4.9 mmol/L

All the CCTA-measurements were significantly higher in the T1DM-group compared to the controls, except mean EAT volume which did not differ between the groups (p = 0.20) (Table 2).

**Table 2:** Coronary plaques, calcification and epicardial adipose tissue in T1DM patients and controls

	Type 1 diabetes (n = 88)	Controls (n = 60)	p-value*
Any plaque, n (%)	75 (85)	28 (47)	< 0.01
CAC, Agatston units	124 (13–671)	1 (0–3)	< 0.01
Calcified plaque volume (mm^3^)	21.0 (1.0–66.0)	0.2 (0.0–7.1)	< 0.01
Mixed/soft plaque volume (mm^3^)	0.0 (0.0–8.7)	0.0 (0.0–0.0)	< 0.01
Total plaque volume (mm^3^)	29.5 (3.9–95.8)	0.4 (0.0–7.4)	< 0.01
Segment involvement score	3 (1–6)	1 (0–2)	0.01^a^
Segment stenosis score	4 (1–8)	1 (0–3)	< 0.01^a^
Epicardial adipose tissue (cm^3^)	52.3 (36.1–65.5)	55 (38.3–79.6)	0.20^a^
Mean EAT attenuation (HU)	− 73.0 (− 76.0 to − 68.8)	− 76 (− 79.4 to − 70.9)	0.01^a^

Presented as median (IQR) unless otherwise stated

*CAC* coronary artery calcification, *SD* standard deviation, *SIS* segment involvement score, *SSS* segment stenosis score, *EAT* epicardial adipose tissue, *HU* Hounsfield units

* Mann–Whitney-U test

^a^Independent samples t-test

**Fig. 1:**
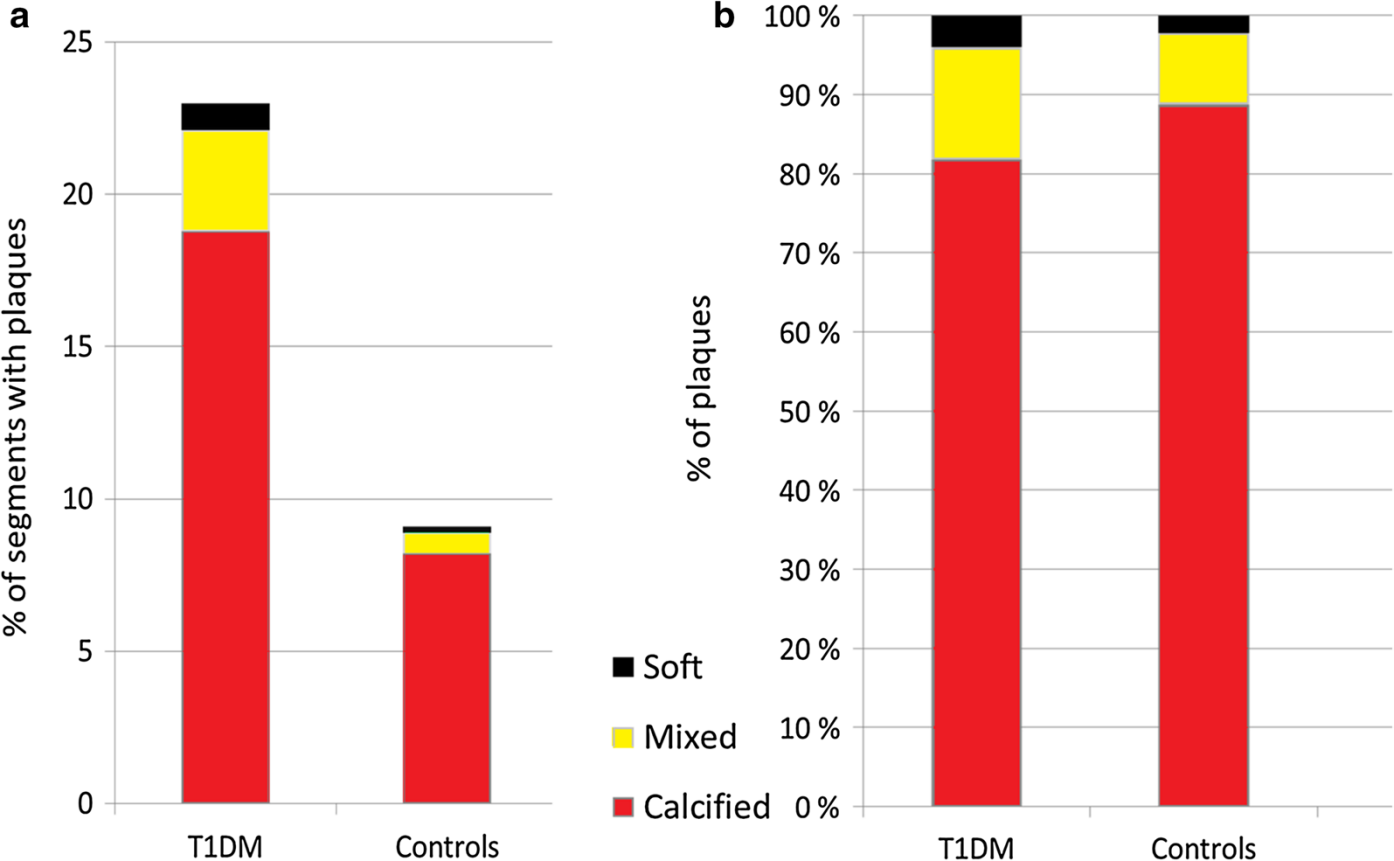
**a** The number of plaques between T1DM and controls. In the T1DM-group, plaques were detected in 23% (324 out of 1408) of the segments, compared to 9.2% (88 out of 960) in the control group (p < 0.01). **b** The distribution of plaque types between T1DM and controls: calcified; 82% vs. 90%, mixed; 14% vs. 8% and soft; 4% vs. 2%, respectively

We detected 23% (324 out of 1408) segments with plaques in the T1DM group; 265 (82%) calcified, 46 (14%) mixed and 13 (4%) soft plaques. In the control group we detected 9.2% (88 out of 960) segments with plaques; 79 (90%) calcified, 7 (8%) mixed and 2 (2%) soft. The distribution of the plaque types are shown in Fig. 1.

In a linear multivariable regression analysis with total plaque volume as dependent variable (Table 3), wLDL-c was the only associated variable after adjusting for sex and age with beta (95% CI) 0.77 (0.19–1.36), p = 0.01.

**Table 3:** Associations between total plaque volume and risk factors for CAD in the diabetes group

	Univariable		Multivariable	
	β (95% CI)	p-value	β (95% CI)	p-value
Age	0.06 (0.00–0.19)	0.05	0.09 (0.00–0.14)	< 0.01
Female sex	− 1.63 (− 2.34 to − 0.91)	< 0.01	− 1.13 (− 1.83 to − 0.43)	< 0.01
wLDL-c	1.00 (0.35–1.64)	< 0.01	0.77 (0.19–1.36)	0.01
wHbA1c	0.44 (− 0.04 to 0.92)	0.07		
wSBP	0.06 (0.02–0.09)	< 0.01		
Waist circumference	0.03 (0.00–0.06)	0.04		

In a multivariable logistic regression analysis with the 25th percentile (n = 21) as dependent variable, the OR (95% CI) were; wLDL-c: 0.18 (0.05–0.70), p = 0.01, wHbA1c: 0.45 (0.20–1.00), p < 0.05, and HDL-c: 0.15 (0.04–0.65), p = 0.01 in an age and sex-adjusted model.

In a sex and age adjusted linear, multivariable regression analysis with log-transformed CAC as dependent variable associated variables were: wLDL-c (beta (95% CI) 0.87 (0.10–1.64), p = 0.03 and pro-BNP (beta (95% CI) 0.005 (0.001–0.010), p < 0.02). In the control group, the only significantly associated variables were female sex and age with beta: − 2.358 (− 3.305 to − 1.412), p < 0.01 and 0.113 (0.044–0.182), p < 0.01, respectively.

The CAC and calcified plaque volume correlated with r = 0.90, p < 0.01.

### EAT volume

The inter- and intraobserver variability of EAT volume was evaluated with an ICC of 0.87 and 0.91, respectively.

No correlations between EAT and CCTA measurements were detected; CAC; r = − 0.04, p = 0.74, calcified plaque volume; r = 0.03, p = 0.77, mixed/soft plaque volume; r = 0.07, p = 0.54, total plaque volume; r = 0.07, p = 0.54 and SIS; r = 0.06, p = 0.59 and SSS; r = 0.08, p = 0.48.

Table 4 shows univariable and multivariable associations between EAT and risk factors for CAD. The only significant association found in the control group was between EAT and waist circumference.

**Table 4:** Associations between epicardial adipose tissue and risk factors for CAD in the diabetes group

	Univariable		Age and sex-adjusted^a^		Multivariable	
	β (95% CI)	p-value	β (95% CI)	p-value	β (95% CI)	p-value
Age	0.20 (− 0.63 to 1.02)	0.635	0.21 (− 0.62 to 1.03)	0.614	0.9 (0.3–1.5)	0.004
Female sex	− 6.15 (− 17.63 to 5.33)	0.290	− 6.23 (− 17.77 to 5.30)	0.286	19.4 (9.4–29.4)	0.000
HDL-c	− 16.06 (− 26.29 to − 5.83)	0.002	− 17.10 (− 28.31 to − 5.89)	0.003	− 9.3 (− 18.1 to − 0.5)	0.038
TG	19.15 (4.97–33.32)	0.009	12.80 (− 0.11 to 25.72)	0.052	− 14.6 (− 27.2 to − 2.0)	0.024
Waist circumference	1.27 (0.93–1.61)	< 0.001	1.54 (0.97–2.11)	< 0.001	1.8 (1.3–2.3)	0.000
wHbA1c	9.14 (2.52–15.77)	0.007	9.12 (2.34–15.89)	0.009		
wLDL-c	7.68 (− 1.66 to 17.01)	0.106				
wSBP	0.083 (− 0.46 to 0.63)	0.763				
Systolic BP	0.18 (− 0.11 to 0.48)	0.214				
Diastolic BP	0.24 (− 0.44 to 0.95)	0.469				
Pulse pressure	0.21 (− 0.15 to 0.57)	0.249				

*HDL-c* high density lipoprotein cholesterol, *TG* triglycerides, *wHbA1c* weighted glycated hemoglobin, *wLDL-c* weighted low density lipoprotein cholesterol, BP; blood pressure, *wSBP* weighted systolic blood pressure

^a^Minimally multivariable model (only adjusted for age and sex)

## Discussion

In this study of patients who have survived more than 45 years with T1DM without a previous diagnosis of coronary heart disease, we found a greater extent and severity of coronary atherosclerosis compared to controls. Plaque volumes, segment involvement score, segment stenosis score and CAC were significantly greater in the T1DM group, but morphological assessments showed mostly calcified plaques (82%). Elevated LDL-c over time was associated with increased plaque volume and CAC. Low LDL-c level and HbA1 over time, in addition to present HDL-c level, was associated with having a more favorable plaque volume (below the 25th percentile ≤ 3.6 mm^3^). The EAT volume did not differ between T1DM and controls. We found no associations between coronary atherosclerosis and EAT volume.

Our study shows a large variation in magnitude of atherosclerotic extent. Interestingly, after more than 45 years of diabetes, 15% have no plaques. As previously reported, 11 (13%) patients were revascularized with PCI or CABG compared to 2 (5%) in the control group [10]. The CAC score also varied substantially between the individuals. We excluded patients with prior cardiac events or known coronary heart disease in order to explore the coronary artery status among asymptomatic long-term T1DM survivors. Therefore, the results are only representative to asymptomatic T1DM patients, without established coronary heart disease. The total burden and characteristic of coronary atherosclerosis in T1DM patient is probably different than in our selected patients, but our study was not designed to investigate it.

In our study, 82% of the plaques were calcified. Soft/mixed plaques have been shown in the MESA-study to be associated with worse outcomes than the more stable calcified plaques [19]. Shemesh et al. investigated the degree of CAC in relation to cardiac events in asymptomatic subjects with and without diabetes [20]. They found that acute events did not occur in subjects with extensive CAC (> 600), but were more likely to occur in subjects with mild or moderate CAC [20]. These results were comparable to findings in the MESA-study; subjects with high CAC (> 400) and very high CAC (> 1000) had equal risk for experiencing cardiac events [21]. Our findings may thereby confirm that calcified plaques represent more stable and long standing atherosclerosis. As shown in a study by Djaberi et al. there are morphologically large differences in plaques between T1DM and T2DM [11]. They found 27% non-calcified plaques in their T1DM-group compared to 65% in the T2DM group. Our participants have a more favorable plaque composition as only 18% of the plaques were defined as mixed/soft plaques. The CAC score in our study was also lower. The patients in the study of Djaberi et al. had shorter diabetes duration (mean of 23 years) compared to ≥ 45 years in our study. The inclusion of long-term survivors of T1DM in our study might explain the discrepancy. Other traditional risk factors were less frequent in our study, which might also contribute to their survival.

The DCCT/EDIC-study described mean HbA1c through 27 years as the strongest risk factor for cardiac events in addition to age in patients with T1DM [22]. In our study, chronic hyperglycemia based on high HbA1c measurements over more than 30 years was associated only with having a low amount of plaque volume (< 25th percentile), while wLDL-c was additionally linearly associated with CAC and total plaque volume. This discrepancy might be due to a higher median HbA1c and a lower median LDL-c level in the DCCT/EDIC-study compared to ours. Patients in the DCCT/ECIT-study were patients with a prior cardiac event, patients that were excluded in our study. The lower HbA1c in our participants may also be a contributing factor for their survival. However, the associations to having the lowest amount of plaque volume suggest that keeping both the LDL-c and HbA1c low over time may have a preventive effect on the development of coronary atherosclerosis. Also, similar plaque characteristics has been described for patients with and without diabetes with elevated HbA1c [23], adding evidence to a role for HbA1c in plaque development. Raised HbA1c is associated with a higher coronary atherosclerotic burden in patients without diabetes [24]. Therefore, we still believe that HbA1c, most likely, plays an important role in plaque development in T1DM-patients. Tinsley et al. also describes a 10 year survival dependent on glycemic control in T1DM patients with > 50 years duration, which further gives evidence to the importance of HbA1c in T1DM patients [25]. A comparison to T1DM patients with a previous cardiac event would be clarifying.

Reducing the LDL-c is the most effective prevention for atherosclerosis in the general population [26]. Statin-use has shown to affect plaque development, observed as cell-death within the lipid cores in addition to the induction of micro-calcifications [27, 28]. These effects are described as plaque-stabilizing, and an inverse linear relationship of plaque density and coronary events are described [29]. Initiation of lipid-lowering treatment is guideline-recommended after 40 years of age in patients with T2DM, but in T1DM statins is recommended only in the presence of microalbuminuria or renal disease [1]. Patients with both type 1 and 2 DM have been shown to be undertreated with statins [30]. In our study, 46% of the T1DM-group reported statin-use, but with a short duration of statin-treatment (2.8 ± 4.3 years). Several publications have shown that high-intensity treatment (LDL-c level target < 1.8 mmol/mL) is required to achieve plaque regression in patients with DM [31, 32]. A higher CAC score has been reported after initiation of statin-treatment due to the conversion in plaque composition [33]. From this one would expect statin-use to have increased the CAC-score in our T1DM group. However, the duration of statin-use is short and the statin-effect cannot be fully evaluated in this cross-sectional study. The low LDL-c-levels and variations in our cohort may be a result of statin-use and accordingly, the findings of non-significant associations to CAC and plaque volume may be explained by a type II error.

Associations between EAT and coronary atherosclerosis are reported by multiple studies [34], suggesting that EAT have a role in the development of coronary atherosclerosis. We did not observe a difference in EAT volume between T1DM-patients and controls, despite a significant difference in coronary atherosclerosis. To our knowledge, EAT has not previously been associated with coronary atherosclerosis in T1DM patients, although associations of coronary atherosclerosis and EAT in patients with T2DM has been revealed [35, 36]. The inconsistent findings between T1DM and T2DM may imply that EAT potentially plays a different role between the types of DM. In T2DM metabolic syndrome, not commonly present in T1DM, has been associated with increased EAT volumes [37]. We did however reveal a strong association between EAT and waist circumference, which implies that visceral fat and fat within the pericardial sac are related. This is consistent with Darabian et al. [12], who found associations of EAT with greater BMI and waist to hip ratio. EAT has been suggested as a new image marker for atherosclerosis, and a lack of association in some patient groups may be important in this discussion. We cannot exclude that the negative associations in our study are a result of a type II error, due to the low amount of mixed/soft plaques.

The influence of glycemic control on EAT volume is unexplored. We did not find associations between EAT and HbA1c. Darabian et al. reported on a significant association between EAT and HbA1c in an age- and sex adjusted statistical model [12]. However, in their study the participants were younger, had a shorter duration of T1DM and a higher BMI compared to our participants. Also, the statistical significance was no longer present after BMI-adjustment. This is similar to our finding, when including waist circumference in the statistical model, the association between EAT and HbA1c was no longer significant.

The use of CCTA in high-risk, asymptomatic patients is debated. Although the radiation hazard and the technical challenge in presence of large calcified plaques are diminished after introduction of newer generation scanners, there is a lack of evidence whether CCTA improves outcomes in asymptomatic patients with diabetes. Muhlestein et al. found no reduction in acute events in their randomized trial [38]. This was also found in the DIAD-study, were patients with T2DM were randomized to myocardial perfusion imaging or not [39]. The identification of patients in the need for further cardiac evaluation is difficult in the absence of symptoms, and other potential selection criteria are warranted. The large variation of presence and extent of coronary atherosclerosis in patients with a long duration of T1DM found in our study supports further evaluation of selection based on other predictors in order to select the right patients for CCTA.

Our study is limited by a small sample size and a cross-sectional design. The control group is also small, and consists of spouses and friends of the patients. Living with a person with diabetes may influence diet and lifestyle, and we cannot exclude that this has affected our results. However, our results are in line with the DanRisk-study of only healthy individuals [40]. The reproducibility of plaque volume is a limitation in CCTA. In our study, most of the plaques detected were calcified, and plaque assessments were performed with a software previously shown to have a high degree of inter-observer variability on calcified and mixed lesions [17]. CAC, however; is an established method with a high degree of reproducibility [41], and our plaque volume score correlated well with CAC.

## Conclusion

In conclusion, patients with a long duration of T1DM have a more extensive and severe atherosclerotic condition, consisting mainly of calcified plaques compared to controls. Maintaining low LDL-c and HbA1c level over time may have a preventive effect on atherosclerotic plaque development, while long-time LDL-c seems to be important for the plaque acceleration in these patients. We found no associations between EAT and coronary atherosclerosis. Larger studies with longitudinal designs are warranted to evaluate the effect of extent and differences of plaque morphology on cardiovascular events in patients with T1DM.

## Abbreviations

BP: blood pressure
CAC: coronary artery calcifications
CAD: coronary artery disease
CCTA: coronary computed tomography angiography
CVD: cardiovascular disease
EAT: epicardial adipose tissue
eGFR: estimated glomerular filtration rate
HbA1c: glycated hemoglobin
HDL-c: high density lipoprotein-cholesterol
HU: Hounsfield units
LDL-c: low density lipoprotein cholesterol
NT-proBNP: N terminal-pro B-type natriuretic peptide
SD: standard deviation
SIS: segment involvement score
SSS: segment stenosis score
T1DM: type 1 diabetes mellitus
T2DM: type 2 diabetes mellitus
TG: triglycerides
wLDL-c: weighted low density lipoprotein cholesterol
wHbA1c: weighted glycated hemoglobin
wSBP: weighted systolic blood pressure
